# Consciousness in active inference: Deep self-models, other minds, and the challenge of psychedelic-induced ego-dissolution

**DOI:** 10.1093/nc/niab024

**Published:** 2021-09-01

**Authors:** George Deane

**Affiliations:** School of Philosophy, Psychology and Language Sciences, The University of Edinburgh, 3 Charles Street, Edinburgh EH8 9AD, UK

**Keywords:** active inference, predictive processing, consciousness, self, psychedelics

## Abstract

Predictive processing approaches to brain function are increasingly delivering promise for illuminating the computational underpinnings of a wide range of phenomenological states. It remains unclear, however, whether predictive processing is equipped to accommodate a theory of consciousness itself. Furthermore, objectors have argued that without specification of the core computational mechanisms of consciousness, predictive processing is unable to inform the attribution of consciousness to other non-human (biological and artificial) systems. In this paper, I argue that an account of consciousness in the predictive brain is within reach via recent accounts of phenomenal self-modelling in the active inference framework. The central claim here is that phenomenal consciousness is underpinned by ‘subjective valuation’—a deep inference about the precision or ‘predictability’ of the self-evidencing (‘fitness-promoting’) outcomes of action. Based on this account, I argue that this approach can critically inform the distribution of experience in other systems, paying particular attention to the complex sensory attenuation mechanisms associated with deep self-models. I then consider an objection to the account: several recent papers argue that theories of consciousness that invoke self-consciousness as constitutive or necessary for consciousness are undermined by states (or traits) of ‘selflessness’; in particular the ‘totally selfless’ states of ego-dissolution occasioned by psychedelic drugs. Drawing on existing work that accounts for psychedelic-induced ego-dissolution in the active inference framework, I argue that these states do not threaten to undermine an active inference theory of consciousness. Instead, these accounts corroborate the view that subjective valuation is the constitutive facet of experience, and they highlight the potential of psychedelic research to inform consciousness science, computational psychiatry and computational phenomenology.

## Introduction

Phenomenal consciousness—the ‘what-it-is-like’ (Nagel 1974) to experience—has now been an area of serious scientific study for at least 30 years (Seth 2018). More recently, the predictive processing framework has generated considerable excitement for its potential contribution to consciousness science. This is largely due to its capacity to go beyond merely positing the presence or absence of consciousness in a given system and to contrastive analysis of the computational mechanisms underlying various phenomenological states. However, a theory of consciousness within predictive processing remains elusive, largely due to the fact that predictive processing is not exclusively concerned with conscious processing (But see: Hohwy 2012; Dolega and Dewhurst 2015; Friston 2018; Solms and Friston 2018; Wiese 2018; Williford *et al.* 2018; Clark 2019; Clark *et al.* 2019; Kirchhoff *et al.* 2019; Whyte 2019; Friston *et al.* 2020; Hohwy and Seth 2020; Ramstead *et al.* 2020b; Safron 2020; Solms 2019, 2021; Whyte and Smith 2020).

Doerig *et al.* (2021) emphasize that predictive processing, as it stands, is insufficiently constrained to provide a theory of consciousness due to being vulnerable to what they call the ‘other systems argument’. A key desideratum for a theory of consciousness on their view is that it ‘should be able to determine which systems, apart from awake humans, are conscious’ (p. 7). They contend that predictive processing fails to deliver on this due to the fact that ‘there is no computational understanding of the crucial characteristics’ (p. 21) that define the conscious condition within predictive processing. In this paper I aim to show that predictive processing, in particular in the recent formulations of the active inference framework, has the resources to deliver a fully-fledged theory of consciousness via a theory of subjectivity grounded in self-modelling.

Active inference is a process theory of the free energy principle (Friston 2010; Friston *et al.* 2017a). Living systems, on this approach, can be understood to embody statistical models of their worlds, where they are biased towards the realization of ‘phenotype-congruent’ outcomes (Ramstead *et al.* 2020a). On this view, agents are ‘self-evidencing’ in that they act in order to maximize the evidence for their own existence (Hohwy 2016). The breadth of explanations within this approach—from microscale explanations applied to understand the adaptive behaviour of bacteria (Tschantz *et al.* 2020) and plants (Calvo and Friston 2017), all the way up to social and cultural dynamics (Veissière *et al.* 2019) and natural selection (Campbell 2016)—brings the need to identify the particular processes associated with consciousness itself into sharp relief.

The structure of the paper is as follows. In ‘The active inference framework’, I give an overview of the relevant mechanics of the active inference framework. In ‘Consciousness in active inference’, I propose a self-modelling theory of subjectivity (Metzinger 2004) within active inference. On this view, consciousness can be understood as arising from ‘subjective valuation’—the system’s inference about precision on control of self-evidencing or allostatic outcomes across multiple levels of the hierarchical generative model. I then consider how subjectivity is ‘shaped’ by hierarchically deep self-models, by considering some examples of disruptions in ordinary self-consciousness. In ‘Consciousness in other systems’, I explore how this characterization can inform attribution of consciousness to other systems, through identification of complex sensory attenuation mechanisms associated with the more elaborate forms of self-modelling understood to underpin consciousness on this account. ‘The selflessness challenge’ considers an objection, the ‘selflessness challenge’—that theories of consciousness that equate consciousness with self-consciousness (‘subjectivity theories’) are challenged by selfless experiences, most notably experiences of ‘ego-dissolution’ occasioned by psychedelic drugs (Letheby 2020; Millière 2020). In ‘Psychedelics and selflessness in active inference’ I build on an existing account of ego-dissolution in the active inference framework (Deane 2020), with a particular focus on the affective and hedonic tone of the experience. In ‘Responding to the selflessness challenge’, I respond to the selflessness challenge; I argue that understanding ego-dissolution in the active inference framework accounts for how the system can still be conscious without the typical structure of experience provided by deep self-models. Instead, these accounts in fact corroborate the view put forward in this paper—that subjective valuation is the constitutive facet of experience. I argue that within active inference, consciousness and subjectivity are best understood in terms of a model of allostatic control. The upshot of this is to show that both the active inference framework and psychedelic science have much to offer on the scientific understanding of consciousness.

## The active inference framework

This section introduces the relevant mechanics of the active inference framework as a formalization of allostasis, before explicating how these mechanics can be understood to underpin phenomenal selfhood. Staying alive requires organisms to maintain homeostasis, an ‘internal balance’ (Cannon 1929), by keeping physiological states—‘essential variables’ (Ashby 2013)—within reasonable bounds. The principles underpinning homeostasis have long been cast within the language of control theory (Conant and Ross Ashby 1970), where homeostasis is achieved through autonomic control loops, such as sweating to lower body temperature. Creatures like human beings exhibit a form of prospective control or ‘predictive regulation’ termed allostasis. In other words, they regulate the internal milieu by anticipating physiological needs and acting to meet them before they arise (Sterling 2012; Pezzulo *et al.* 2015; Stephan *et al.* 2016; Schulkin and Sterling 2019; Corcoran *et al.* 2020).

The active inference framework formalizes allostasis in terms of a single imperative—to minimize the divergence between expected and observed outcomes under a generative model that is fine-tuned over the course of phylogeny and ontogeny (Badcock 2012). On the free energy principle, the basic imperative is to remain in ‘expected’ states—a ‘species-specific window of viability’ (Clark 2013, 13). The free energy principle (Friston *et al.* 2010; Badcock *et al.* 2019) thus casts control theoretic ‘essential variables’ in terms of high precision prior expectations. This means that organisms are phylogenetically endowed with an expectation (and therefore bias to act) to e.g. maintain a body temperature within reasonable bounds. These high precision prior expectations are not amenable to perceptual revision and instead must be fulfilled through corrective action (e.g. seeking out a shaded tree to maintain viable body temperature). Here, prior expectations are acquired through past experience, over the course of both phylogeny and ontogeny (Badcock *et al.* 2019).

The notion of a hierarchical generative model lies at the centre of the active inference framework. A generative model is specified in terms of probabilistic beliefs about how observations relate to the states of the world that cause them (the likelihood), beliefs about how the states evolve over time and prior beliefs—beliefs about the state of the world prior to observation. Inference here corresponds to inversion of the model in computing the probability of the unknown or hidden causes of the impinging sensory signals. It is intractable to compute this posterior directly and so approximate Bayesian inference is made tractable by optimization of a posterior although variational inference. In this way, the approximate posterior converges towards the true (unknowable) posterior through the minimization of variational free energy (Friston *et al.* 2017a). Crucially, free energy can also be interpreted as a bound on the evidence for a generative model. This means that minimizing the free energy just is maximizing model evidence—hence the notion of self-evidencing (Hohwy 2016; Palacios *et al.* 2020).

Active inference formalizes allostasis (and action) as an inference problem, ‘planning as inference’ (Kaplan and Friston 2018) under the free energy principle (Friston 2019). The planning and execution of action or a sequence of actions (a ‘policy’)—under this scheme—becomes a problem of inference where the action with the highest prior probability is that which minimizes ‘expected’ free energy—the expected dyshomeostatic consequences of an action policy (Kaplan and Friston 2018). Selection of optimal action policies involves having counterfactually rich expectations of the states of affairs that would be brought about contingent on actions (Seth 2014; Pezzulo 2017; Friston *et al.* 2017a). In other words, to select action policies that maximize self-evidencing outcomes over time, organisms rely on deep temporal models (Friston *et al.* 2017a). Deep temporal models encode expectations about the evolution of states of affairs over time contingent on action policies, such that the system can infer actions that result in sensory states conducive to continued existence—sometimes called the ‘attracting set’ (Friston 2012).

Just as with perceptual inference (belief updating or ‘state estimation’), action selection is understood in terms of Bayesian model selection, where possible action policies are scored with respect to the expected free energy associated with pursuing a given policy. Here, the agent is equipped with beliefs about transitions between (inferred) states of the world, where beliefs about state transitions are updated in light of the action or action policy that is currently being pursued. Conditioning state transitions on actions—in the generative model—allows the agent to select action policies that have the least expected free energy, where expected free energy can be decomposed into ‘epistemic and pragmatic’ value, such that the agent can learn about its environment while realizing prior preferences (Friston *et al.* 2015; Friston *et al*. 2017). This process of selecting actions that minimize expected free energy, which has been dubbed ‘allostatic control’ (Kiverstein and Sims 2021), is not itself thought to underwrite phenomenal selfhood (and experience). Rather, I will argue that phenomenal selfhood is underwritten by hierarchically deep inference about the precision on expected free energy. This ‘allostatic control model’—understood as a ‘subjective valuation’ on the ‘fit’ of the action model to the world spanning multiple timescales—will be unpacked in sections to come. First, it is worth unpacking what it is the system needs to control on this account—the minimization of expected free energy—in terms of the realization of prior preferences and maximization of epistemic value.

### Pragmatic value and prior preferences

Agents are not disinterestedly inferring their control of sensation via action. Rather, in active inference the agent acts to realize prior preferences—changing the world to make it conform to prior expectations, as opposed to changing beliefs to conform to the world (i.e. perceptual inference). Active inference thus recasts ‘essential variables’—physiological quantities that must remain within specific bounds for an organism to stay alive (Ashby 2013)—as high precision ‘prior preferences’. Prior preferences are phenotype-specific states that the organism expects itself to be in—connecting control to states of the body and views of selfhood based in interoceptive inference (Seth and Friston 2016; Barrett 2017; Seth and Tsakiris 2018). Prior preferences about essential variables encode probability distributions over states (rather than a single ideal setpoint), and the sufficient statistics that specify this setpoint (mean and precision) are free to vary and can be toggled according to the context (Ainley *et al.* 2016). This is key in allostasis, as it allows for temporary deviations from a homeostatic setpoint in order to realize sensory states in the ‘attracting set’ on longer timescales. For instance, heart rate and blood pressure are more flexible to contextual alteration in order to realize certain actions (e.g. fleeing from a predator), while others such as blood pH and core body temperature may be less variable due to more constant high precision (Corcoran *et al.* 2020). While many prior preferences are phylogenetically endowed, over the course of ontogeny an organism will acquire prior preferences that subtend increasingly deep temporal scales. The expected free energy of a given policy, then, is going to depend to some degree on how much the given policy fulfils prior preferences, and so, as we will see, a critical part of the phenomenal self-model is understood in terms of an inference about control of the realization of prior preferences.

### Epistemic value

Self-evidencing agents not only act in order to realize prior preferences, but they also engage in novelty-seeking behaviours that realize epistemic value (Friston *et al.* 2015, 2016). The epistemic value or affordance of a given policy refers to the information gain or resolution of uncertainty about the causes of sensation. Optimal epistemic action, or ‘epistemic foraging’, requires the agent to have beliefs about their own uncertainty, enabling action directed towards higher sensory precision. Agents minimizing expected free energy seek out observations that resolve ambiguity about the state and causal structure of the world. Curiosity and novelty-seeking behaviour are accounted for within this formulation of epistemic action (Friston *et al.* 2015; Kiverstein *et al.* 2017; Mirza *et al.* 2018; Pezzulo and Nolfi 2019). This can be understood in terms of sensitivity to long-term epistemic affordances (Bruineberg and Rietveld 2014; Parr and Friston 2017). When the agent is confident about its model of the world, and epistemic value is much the same across policies, pragmatic or instrumental value (fulfillment of prior preferences) dominates behaviour.

## Consciousness in active inference

In this section I argue for a self-modelling theory of subjectivity within the active inference framework. The central claim is that consciousness is best understood as being underpinned by hierarchically deep self-models understood in terms of ‘subjective valuation’—precision estimation on the action model—across multiple levels of the generative model. In other words, consciousness arises in a system that evaluates the ‘fit’ of its model with the world—how well outcomes align with expected outcomes given the kind of creature it is—across multiple timescales. This inference informs action and policy selection across multiple timescales—from refining a motor action based on proximal sensory outcomes to domain general inferences about expected control across variable environments on longer timescales. The upshot of this section is a view of subjectivity as ‘subjective valuation’, understood as a kind of self-modelling that is (in normal awareness) intimately bound up with aspects of the phenomenal self, such as the sense of agency and affectivity.

For clarity about how different contents of consciousness map to the generative model, I sequentially unpack the inferential architecture of the hierarchical generative model and how this architecture relates to conscious contents in the following four subsections: ‘Perception’ covers basic models of perception as (hidden) state estimation approximating Bayesian inference; ‘Precision’ covers precision-weighting in perceptual inference; ‘Active inference’ connects active inference and planning as inference as a formalization of allostasis (Attias 2003; Botvinick and Toussaint 2012; Kaplan and Friston 2018; Millidge 2020) to particular aspects of phenomenal selfhood, arguing that an inference about ‘agentive control’—endogenous control of self-evidencing outcomes via action—means that phenomenal selfhood is implicit in the active inference framework. ‘Affectivity’ extends this picture to argue that affective inference can similarly be understood as an inference about allostatic control, manifesting computationally as precision on the action model that guides domain general policy selection and is closely related to interoceptive and emotional inference (Seth and Friston 2016; Fotopoulou and Tsakiris 2017; Smith *et al.* 2019). In ‘Towards a theory of subjectivity’ I argue that this hierarchically deep inference about endogenous control is not merely contents of consciousness, but rather ‘shapes’ subjectivity, permeating perception of the world (and self). This functions to ‘tune’ organisms to opportunities for (adaptive) action in the environment.

### Perception

A simple starting point to think about the generative model is to consider it in the case of moment-to-moment perception, which can be understood as state estimation or the brain’s ‘best guess’ of the hidden causes of the incoming sensory signal. This basic (illustrative) generative model maps the relationships between observations (o)—the incoming sensory data and the hidden states (s) in the world that caused the sensorium. A ‘likelihood mapping’—encoding the probability of an observation under a generative model, given its causes (the hidden states in the world)—captures this relationship. In other words, what is the probability of the observations ‘given’ the world is in a certain state? Formally, this is denoted as a likelihood model P(o|s). The full generative model includes beliefs about the most likely state of the world prior to any observation, which is known in Bayesian inference as the ‘prior’, denoted by P(s).

Of course, what the system needs to infer is not the probability of observations given the hidden states, but the inverse, the probability of hidden states given the observations. This is achieved through variational Bayes—movement from what the system has access to observations, prior beliefs and beliefs about how observations are caused by hidden states, to what it needs to infer: the hidden states that are the most probable causes of the incoming sensation—i.e. the posterior probability of the states ‘given’ the observations P(s|o). Inference about hidden states given the observations is known as model inversion, as it is the inverse mapping from the consequences or outcomes to causes. Model inversion finds the most plausible cause of observations, and as such, perception can be understood as ‘posterior state estimation’, i.e. estimating the hidden states and other variables that cause sensory outcomes. Formally, the process of updating a prior belief into a posterior belief on the basis of new sensory evidence is called Bayesian belief updating.

### Precision

Predictive processing architectures benefit from radical contextual flexibility afforded by ‘precision-weighting’. Precision-weighting can be understood in terms of amplification or gain control, regulating the interaction between top-down and bottom-up signals by weighting them according to their expected ‘precision’—where heavily weighted priors or prediction errors exert greater influence in determining the resulting posterior inference. Precision can be understood as a prediction of the reliability of one’s own beliefs, —e.g. confidence in the likelihood mapping. Attentional processes operate on second-order statistics like precision: in other words, attentional states can be understood in terms of beliefs about the (precision of the) system’s beliefs. This means that precision—on the simple generative model just described—can be understood as the extent to which the system thinks observations reliably map to hidden states. Formally, precision is the inverse variance of a probability distribution (Feldman and Friston 2010), and optimization of precision-weighting is frequently equated to attention within predictive processing schemes (Feldman and Friston 2010; Clark 2013). Heuristically, precision can be thought of as predictability or reliability of predictions—something that itself has to be inferred.

Predictive processing offers a picture of how a confluence of precision-weighted informational streams determines perceptual inference. Perceptual inference involves integrating information from across modalities to infer the hidden causes of sensation—for instance, during binocular rivalry, auditory (Lunghi *et al.* 2014), olfactory (Zhou *et al.* 2010) and tactile information (Lunghi and Morrone 2013) have all been shown to influence which percept is dominant. Interoceptive (Salomon *et al.* 2016) and proprioceptive (Salomon *et al.* 2013) information have been shown to affect visual experience using a continuous flash suppression paradigm.

Cue integration is one such example of how integration of (precision-weighted) informational streams gives rise to the resultant percept. In a cue combination task, observers are presented with two more cues about a perceptual variable—such as, in early cue integration, the use of two depth cues (e.g. stereo and motion parallax) (Landy *et al.* 1995). The reliability of the cues can be varied (for instance, by varying their visibility or contrast) to make one cue more reliable than the other. A series of studies have shown that when observers are asked to indicate their percept (a depth estimate), their estimates—depending on the cues, weighted by their precision, in combination with a prior probability—are approximately Bayes optimal (Landy *et al.* 1995; Ernst and Banks 2002; Knill and Pouget 2004).

Another example is gist perception in object recognition—where the ‘gist’ of the scene engages past experience to generate the most likely prediction about the object’s identity (Bar 2003; Oliva and Torralba 2006). For instance, in the case of an ambiguous object, the context of a scene can determine whether the ambiguous input is perceived as a hairdryer or a drill depending on whether the context is in a bathroom or a workshop. These predictions are fed back to early visual areas to speed perception by constraining the hypothesis space of possible interpretations.

‘Predictive penetration’—social, cognitive and emotional—have all been demonstrated (O’Callaghan *et al.* 2016). Sensorimotor contingencies—predictions about how the world changes according to our actions—have also been shown to shape the contents of consciousness (Skora *et al.* 2021). Importantly, precision is thought to mediate engagement with affordances—latent possibilities for action (Cisek 2011; Pezzulo and Cisek 2019) and sensory attenuation—the top-down filtering out of afferent (incoming) information, both from the body (interoception) and the senses (exteroception). As we will see, striking the right balance of precision in perceptual inference requires deep self-models.

The basic predictive processing story so far casts the brain as a hierarchical prediction machine using belief updating schemes to approximate Bayesian inference by utilizing ‘priors’ (probability distributions about hidden states of the world) and incoming sensory data (‘prediction errors’) to arrive at a posterior estimate: a ‘best guess’ of the hidden causes of sensory signals (Clark 2013, 2015; Hohwy 2013; Aitchison and Lengyel 2017; Nave *et al.* 2020). Prediction errors are a central concept in predictive processing and the free energy principle. In a mathematically general sense, the free energy gradients that drive Bayesian belief updating in the free energy principle can always be expressed as a prediction error (in the form of a difference in log probabilities). In specific schemes, such as predictive coding, prediction errors are often treated as explicit variables that may be encoded by the activity of specific neuronal populations in the brain (e.g. superficial pyramidal cells).

Predictive processing delivers a compelling story about the contents of perception, where ‘conscious perception is determined by the prediction or hypothesis with the highest overall posterior probability—which is overall best at minimizing prediction error’ (Hohwy 2012, 4). As yet, however, it is not clear why there should be ‘something-it-is-like’ to perceive—i.e. how subjectivity itself is underwritten by the mechanism’s prediction error minimization. In the next two sections, we see how these same principles can be built up to give an account of a subject of experience in terms of phenomenal self-models.

### Active inference and the phenomenal self

‘The active inference framework’ gave an overview of active inference and allostatic control. This section and the next section on affective inference will look at how this notion of how allostatic control can be tied to phenomenal selfhood, in particular how the system’s inference or evaluation of its own allostatic control—used to inform ongoing policy selection across multiple timescales—can be understood to underpin the phenomenology of being an agent.

The phenomenal self-model can be understood as **‘**the content of the conscious self: your current bodily sensations, your present emotional situation, plus all the contents of your phenomenally experienced cognitive processing’ (Metzinger 2003, 299). Phenomenal selfhood is understood as being ‘The way you appear to yourself, subjectively, consciously’ (Metzinger 2004, 26). Increasingly, the formal principles of self-modelling implied by active inference are thought to underpin phenomenal self-modelling (Limanowski and Blankenburg 2013; Hohwy and Michael 2017; Friston 2018; Limanowski and Friston 2018, 2020; Deane 2020; Deane *et al.* 2020). On these views, ‘some notion of “self-hood” or “self-agency”—in the sense of inference about control—is inherent in active inference’ (Limanowski and Friston 2020, 2), as optimal action planning rests on this notion of control—where the system infers its control of sensation via action to realize self-evidencing outcomes. As such, the self is seen as being a ‘hypothesis or latent state (of being) that can be associated with a self-model’ (Limanowski and Friston 2020, 3). In what follows, the inference about precision on intentional selection—cast in terms of an ‘allostatic control model’—is thought to underpin phenomenal selfhood. This inference about allostatic control is understood to span from lower-level motor control all the way up to domain-general (and abstract) expectations of allostatic control across contexts.

Inference about the control of sensation via action—‘agentive control’ (Deane *et al.* 2020)—has been linked to the phenomenology of being an agent (Limanowski and Friston 2020). Agentive control is best understood as the system’s inference of its own ability to endogenously control sensory inputs via action (Hohwy and Michael 2017) and as such is intimately related to the ‘sense of agency’—the experience of oneself as an agent who can cause events by acting (Haggard 2017). Agentive control is understood here to be temporally deep, because expectations of the consequences of actions are not confined to the immediate future, but can predict abstract and distal outcomes (Pezzulo *et al.* 2015). For example, an agent may expect proximal sensory consequences of tipping a watering can to water a plant pot, but also have temporally deep—and ‘abstract’ (Gilead *et al.* 2019)*—*expectations about the form of the plant over the timescale of weeks and months contingent on actions. Recall that, under active inference, lower levels of the predictive hierarchy track regularities that are unfolding on shorter timescales and higher levels track regularities unfolding on longer timescales (Kiebel *et al.* 2008; Friston *et al.* 2017b). In just the same way that an organism can infer its own ability to control the immediate sensory consequences of action, by tracking regularities over time it can track its control of sensory outcomes more generally, where expectations of the downstream consequences of action inform policy selection (Friston 2018).

A central idea here is that, in acting, the system must infer ‘itself’ as able to bring about the (self-evidencing) consequences of the action, where the self-evidencing consequences are understood in terms of expected free energy. Inference about agentive control is intimately related to the allocation of precision and most specifically the lowering of precision to attenuate sensory evidence in certain contexts. There are two forms of sensory attenuation—‘physiological’ and ‘perceptual’ sensory attenuation (Palmer *et al.* 2016)—that critically relate to agentive control on this account.

The first to consider is ‘physiological sensory attenuation’ (Palmer *et al.* 2016), which is critical for movement initiation in active inference (Brown *et al.* 2013). Action initiation involves ‘systematic misrepresentation’ (Wiese 2017)—whereby proprioceptive evidence that, for instance, my arm is not moving, is attenuated to allow the system to bring about the desired movement (Adams *et al.* 2013; Brown *et al.* 2013). Higher-level prior beliefs attenuate current sensory evidence and higher precision is afforded to the anticipated sensory consequences of the desired action. Prediction error is then suppressed by making the prediction come true, through reflex arcs at the lowest level of the hierarchy (Parr *et al.* 2018). In the setting of motor control, this perspective on action is closely related to ideomotor theory (Limanowski 2017) and 20th-century formulations in terms of the equilibrium point hypothesis (Feldman and Levin 1995). In other words, all that is required for intentional movement is a specification of the desired sensorimotor endpoint of a movement—and motor reflexes bring the position of the body into line with that equilibrium or setpoint. Another perspective on this formulation is perceptual control theory (Mansell 2011), where action is in the game of bringing about desired sensory consequences—in this instance proprioceptive sensations from the musculoskeletal system. In order to move, then, the system predicts itself in the desired state, and as able to control sensation via to bring about the desired state. Physiological sensory attenuation thus aids in entertaining counterfactual hypotheses about oneself (Limanowski and Friston 2020) in order to generate the self-fulfilling prophecy of moving.

This self-attenuation needs only be applied transiently for movement initiation, but these same sensory attenuation mechanisms are thought to underpin various states of altered self-experience. For instance, in the ‘rubber hand illusion’ (Botvinick and Cohen 1998), visual information about the location of the hand is deemed to be precise due to the corroborating synchronous stroking pattern on the rubber hand, while the conflicting proprioceptive input suggestive of the hand’s real location is down-weighted in order to maintain a coherent bodily representation (Limanowski and Friston 2020).

‘Perceptual sensory attenuation’ is the top-down filtering of afferent information to limit how much feedback is received from self-generated movement. On the current account, perceptual sensory attenuation is critical to the formation of the ongoing inference about agentive control. Originally developed as a theory of motor control, the ‘comparator model’ posits that motor commands are refined through comparing sensory consequences of an action with the intended consequences of an action (Miall and Wolpert 1996; Wolpert and Flanagan 2001). Subsequently, the comparator model has been used to account for the sense of agency (Feinberg 1978; Frith 2005; David *et al.* 2008), where inference about endogenous control over the causes of sensory signals is thought to underpin the sense that an action is agentive or self-generated. For instance, sense of agency would be low in the case of a mismatch between motor output and sensory input, such as (when wearing a VR headset) a virtual hand that moved in a way that did not correspond to movements of the subject’s real hand. The mismatch between the expected and actual consequences of a given action justifies the attribution of sensory outcomes to exogenous (external) rather than endogenous (internal) causes, such that attribution of sensory outcomes to exogenous causes results in a reduced or absent sense of agency (Sirigu *et al.* 1999). Indeed, incongruent action-outcomes have been linked to a reduced sense of agency (O’Sullivan *et al.* 2018). A self-other distinction critically relies on balancing this attribution to exogenous and endogenous causes.

Selectively attenuating precision on sensory inputs allows the system to filter out irrelevant inputs (Crapse and Sommer 2008a), such as those caused by self-generated actions. One such example of this is saccadic suppression, where, despite saccadic eye movements, perception of the environment remains stable. Reduced precision on afferent inputs from self-produced tactile sensation is thought to cause inability to tickle oneself (Blakemore *et al.* 2000). Sensory attenuation in relation to movement and self-experience will be discussed in more detail in the sections on disturbances of self-consciousness and other minds.

### Affective inference

Affective inference, on the current account, is also understood in terms of an inference about allostatic control. Affective inference—in terms of a contextually flexible inference of the precision on prior preferences and epistemic affordances (opportunities for epistemic gain or uncertainty reduction)—acts to ‘tune’ the organism to possibilities for self-evidencing action in the environment. Precision on prior preferences is inferred across the control hierarchy (or ‘deep goal hierarchy’ Pezzulo and Cisek 2019). Pain perception is a great example of ‘tuning’ to the current context. Precision is allocated to, for instance, the ‘healthy body condition’ prior preference (Ongaro and Kaptchuk 2019) according to a host of contextual factors. This flexibility enables organisms to ‘tune their own pain perception according to both their prior beliefs and the specific biological goals they believe are attainable in that context’ (Moutoussis *et al.* 2014, 70). In accordance with this, mounting evidence speaks against the more classical view of pain as tracking tissue damage, in favour of a view of pain perception as underpinned by a process of inference. In particular, Bayesian models of pain perception provide evidence that affectively charged percepts are inferential in nature (Morton *et al.* 2010; Anchisi and Zanon 2015). For example, studies show that patients who receive treatment in a medical context experience considerably higher pain relief than those who receive analgesic drug treatment covertly (Benedetti *et al.* 2003, 2011). The felt intensity of pain can be adjusted according to the context and the survival needs of the animal, modulated by attention, expectation, conditioned pain modulation and placebo responses (Atlas and Wager 2012; Atlas *et al.* 2014; Kirsch *et al.* 2014; Kong and Benedetti 2014). Even social information can have a profound influence on experience: other people’s pain reports affected participants’ pain experience and physiological indicators of increased pain such as the skin conductance response (Koban and Wager 2016).

Inference about endogenous control of self-evidencing outcomes can thus be understood as an inference about ‘subjective fitness’—the expected precision of the organism’s phenotype-congruent action model (Hesp *et al.* 2021). On this account, interoceptively registered bodily changes track how well the organism is doing at minimizing expected free energy—i.e. fulfilling prior preferences and resolving uncertainty (Joffily and Coricelli 2013; Seth and Friston 2016; Kiverstein *et al.* 2019, 2020). This contextually flexible evaluation of model fitness is essential for organisms to persist and perform adaptive actions in volatile environments. Promoting self-evidencing outcomes on longer timescales requires organisms to be sensitive not only to prediction error reduction in the present, but the rate of prediction error reduction over time (Joffily and Coricelli 2013; Kiverstein *et al.* 2019; Van de Cruys 2017). On this view, certain rates of prediction error over time—such as progress towards a goal—become prior preferences fulfilled by (temporally extended) action. As such, deviation from the prior preference manifests to the system affectively, acting as motivation to realize the prior preference via action. The roots of these approaches can be traced to control theoretic precursors that postulate a second feedback system that senses and regulates the rate of the action guiding system (Carver and Scheier 1990).

Inference about the reliability of the action model allows the system to increase or decrease precision on the current policy (Kiverstein *et al.* 2019; Hesp *et al.* 2021). For instance, if the current policy is reducing prediction error at a rate that is worse than expected, this manifests to the system as negative affect and acts as an incentive to discontinue the current course of action. Affective valence here is being reimagined within the active inference framework as a ‘domain general controller’ (Deane *et al.* 2020; Ramstead *et al.* 2020b). Inference about how well the system can expect to reduce error via action ‘in general’ is informative as it informs precision on policies across contexts, acting as a domain general prior on the precision of policies generated by the action model (Hesp *et al.* 2020, 2021).

Deane *et al.* (2020) suggest that a sensitivity to worse than expected rates of prediction error reduction over time (Kiverstein *et al.* 2017, 2020; Hesp *et al.* 2021), manifesting phenomenologically as negative affect, drives the system to switch to more tractable goals. For instance, while loss of control in a particular context (such as learning to play a particularly difficult piece in a piece of music) might create negative affect, this negative affect functions as an incentive to switch to a task with a better expected rate of prediction error reduction. As such, loss of control in a particular domain does not necessarily impact a more domain-general sense of control, related to more fundamental and pervasive sense of self as a causally efficacious agent. As such, affective inference—inferring precision on prior preferences and epistemic affordances across multiple hierarchical levels—tunes the organism to adaptive actions in the given context.

### Towards a theory of subjectivity

In his paper ‘What is it like to be a bat?’ Thomas Nagel argues:

An organism has conscious mental states if and only if there is something it is like to ‘be’ that organism—something it is like ‘for’ the organism. (Nagel 1974, 436)

The preceding sections saw how the sense of being a ‘self’ is inherent in active inference. It may be argued however that while the active inference framework may shed light on the computational correlates of the contents of consciousness, it cannot shed light on why there is a subject of experience in the first place—why there is ‘something it is like’ to be an agent.

Why then, in the active inference framework, is experience ‘felt’? The claim here is that phenomenal consciousness is underpinned by estimation of the precision of its own action model. To be more specific: the system needs to engage in subjective valuation, i.e.*—*set precision on competing action policies across multiple levels of the hierarchy based on inference about endogenous control of self-evidencing outcomes. Precision on action policies on this account is understood to be a fundamentally ‘affective inference’. This gives a novel perspective on how to conceive of why incoming sensory data should mean something ‘for me’ as a subject, computationally the meaning being ‘What does this mean for precision on my action model?’. This confidence estimate in the action model spans multiple levels of the hierarchy, from the highest levels underpinning the most invariant expectations of allostatic control across contexts to the lowest levels tracking moment-to-moment sensorimotor action selection and correction. This valuation is ‘subjective’ in the sense that it can be out of step with reality—i.e. the system could be over-confident or under-confident in these estimations.

As such, the mechanisms underpinning phenomenal consciousness on the current picture—a ‘deep control model’—act to ‘tune’ the organism to adaptive action in the world across multiple interlocking timescales. On this view, our status as ‘beast machines’ shapes our subjective experience (Seth and Tsakiris 2018). For instance, it has been demonstrated that neutral stimuli are more often perceived as fearful when subjects were given (false) feedback of increased heart rate (Anderson *et al.* 2012). Hierarchically (and temporally) deep contextualization of interoceptive signals tunes an organism to appropriate action and engagement with environmental affordances (Pezzulo and Cisek 2019) and assigns appropriate weight to priors and ascending prediction errors across the cortical hierarchy. Notice that this means that even state estimation associated with perceptual inference is determined by the overarching inference about control of self-evidencing outcomes—both in terms of the predictive models encoding sensorimotor relations (‘counterfactual richness’) grounding the subjective reality of perceptual contents (Seth 2014) and in terms of those perceptual contents being filtered through deep goal hierarchies (Pezzulo *et al.* 2015, 2018).

It is through this inference about allostatic control that a conscious agent encounters ‘a structured world apt for action and intervention, and inflected at every level, by an interoceptively-mediated sense of mattering, reflecting “how things are for me as an embodied agent”’ (Clark 2019, 7). The ‘sensorimotor’ and ‘affective’ dimensions of consciousness on this view are both underpinned by a hierarchically deep inference on the fit between the actual and expected outcomes of actions that informs subsequent action selection. Even the low-level inference about sensorimotor control operates within the context of nested goal hierarchies and as such is understood as inherently affective in virtue of being enslaved by higher-level goals (Pezzulo *et al.* 2015). For instance, failing to execute a motor command correctly involves violating an expected rate of prediction error reduction. Hierarchically deep inference means that experience of the world is suffused with our ‘cares and concerns’ (Ramstead *et al.* 2020b) across multiple levels and accords with the view that visual perceptual experience is determined by the agent’s ‘poise’ over the ‘action space’, where we encounter the world as a ‘matrix of possibilities for pursuing and accomplishing one’s intentional actions, goals and projects’ (Ward *et al.* 2011, 1).

Interpreting what the current sensory input means ‘for me’—i.e. for precision on the action model at different levels of the hierarchy—allows the system to arbitrate between competing affordances on different timescales. The upshot of this is a common motivational currency for navigating trade-offs between affordances competing on different timescales (Pezzulo and Cisek 2019). Conceiving of valence as a ‘common currency’ to arbitrate between action plans in this way connects this proposal to numerous accounts of phenomenal consciousness in the literature (Cabanac 1992; Morsella 2005; Merker 2007). Moreover, contextual modulation of the precision on expected free energy is critically related to flexible behavioural control (Pezzulo *et al.* 2015) and as such bridges the current story to the association between consciousness and flexible behaviour (Dehaene *et al.* 2017).

### The shape of subjectivity: disruptions in (self-)consciousness

Altered self-experience provides some of the most compelling illustrations as to how subjectivity is shaped through an inference about allostatic control. This section briefly considers depersonalization and meditation.

A domain-general loss of precision control has been used to understand depersonalization disorder (Deane *et al.* 2020). This account—through connecting views of affectivity in terms of precision estimation on expected free energy to the feeling of being an agent—casts the computational mechanisms of depersonalization as an inferred loss of allostatic control, whereby the system posits itself as causally inefficacious at realizing self-evidencing outcomes across contexts. As we saw in the previous section, the affective system usually acts to tune the system to action opportunities across multiple interlocking timescales. Depersonalization is understood as occurring due to a global loss of precision on action policies and as such the world loses ‘phenomenal depth’—as described by sufferers of depersonalization—in that it ceases to solicit engagement and is perceived as flat or two-dimensional (Medford *et al.* 2006; Ciaunica *et al.* 2021). Major depression, similarly, has been characterized in terms of ‘domain general inference of a loss of allostatic control’ (Ramstead *et al.* 2020b).

This phenomenology is contrasted with a perceived gain in allostatic control in meditation practitioners, as precision (on prior preferences, for instance) becomes increasingly under endogenous control (Deane *et al.* 2020). This account makes use of the fact that mental action in active inference follows just the same principles as the account of action initiation put forward earlier, but where the hidden states are ‘attentional’ states (precisions) and the state transitions are transitions between attentional states (Smith *et al.* 2020). The idea here is that ‘focused attention meditation’ (Lutz *et al.* 2019) can be understood as the endogenous withdrawal of precision from prior preferences, due to the practice of repeatedly bringing attention back to the attentional object, such as the breath. For instance, the sensation of an itch can be understood in terms of increased precision on a scratching policy. Through withdrawing precision from the sensation and back to the target sensation (such as the breath) the system learns an extra level of agentive control, i.e.—endogenous control of precision on prior preferences. Over time, this becomes domain general, such that the system learns to have precision control over its own affective system.

## Consciousness in other systems

This section will provide a preliminary sketch of how, through identifying the neural mechanisms associated with the allostatic control model, we can assuage the concerns posed by the ‘other systems’ argument and begin to make inferences about which kinds of creatures and systems are likely to be conscious subjects. The view put forward in this paper is that inference about confidence in endogenous self-evidencing capacity, or precision on expected free energy, underpins phenomenal consciousness. Inference about control balances physiological and perceptual sensory attenuation mechanisms in order to ‘tune’ organisms to act adaptively in their environment, enabling both adaptive motor control and determining the perceptual salience in the given context. This section sketches how the complex sensory attenuation mechanisms associated with consciousness on the present account can give clues as to the neuroanatomical substrates and processes that are indicative of conscious experience.

Holst and Mittelstaedt (1950) identified an interpretative problem as to whether sensory signals arise from the environment or the animal’s own muscles and movement, dubbed the ‘reafference problem’. The reafference problem arises due to the fact that sensory receptors are indifferent to the cause of their activation, whether it be from exafference—activation stemming from the environment, or reafference*—*inputs that result from an animal’s own movements (Holst and Mittelstaedt 1950). Sensory neurons are able to respond with high sensitivity to exafferent inputs despite disruptive self-generated inputs (Bell 1981; Poulet and Hedwig 2002; Eliades and Wang 2008; Keller and Hahnloser 2009; Ahrens *et al.* 2012). Across species, the sophisticated filtration process underpinning this high sensitivity to exafferent inputs is thought to be achieved through the mechanisms of ‘corollary discharge’—predictions of the sensory consequences of actions that act to suppress reafferent inputs (Crapse and Sommer 2008a). In predictive processing, corollary discharge can be understood simply as top-down predictions that explain away sensory prediction error (Friston *et al.* 2010).

Crapse and Sommer (2008a) make a distinction between lower-order (reflex-inhibition and sensory filtration) and higher-order (sensory analysis and sensorimotor learning/planning) corollary discharge based on their underlying neuroanatomical substrates. Lower-order corollary discharge enables reflex inhibition and sensory filtration, in order to regulate and control sensation entering the central nervous system, and appear to have the function of ‘transient, protective inhibition of sensory networks’ (Crapse and Sommer 2008a, 592). For example, the nematode *Caenorhabditis* *elegans*—often used to study simple nervous systems—has a simple behavioural repertoire and only 302 neurons and uses lower-order corollary discharge in order to inhibit reflexes that would be triggered by reafference. Klein and Barron (2016) argue that in this very simple nervous system—with only two layers separating sensory neurons from motor neurons—there is no evidence that this sensory attenuation mechanism contributes to a structured model of the self or a model of action-outcome contingencies informing selection from a range of possible actions. This is behaviourally as well as neuroanatomically apparent: when hungry, nematodes respond with increased locomotion in a random search pattern (Lüersen *et al.* 2014; Artyukhin *et al.* 2015). By contrast, hungry rodents, ants and bees will direct their search towards locations where they have encountered food previously (Oades and Isaacson 1978; Seeley 1995; Wehner 2013). In the case of the nematode, the corollary discharge does not seem indicative of a model of temporally deep control, and the lack of anticipatory and goal-driven behaviour makes it unlikely for nematodes to have phenomenal consciousness on the present proposal.

Crapse and Sommer (2008a) identify higher-order corollary discharge as involved in predictive control in perceptual cohesion and action sequencing—this ‘does’ seem suggestive of a deep control model. For example, bats explore their environment by emitting beams of sound and then comparing the emission with the spatiotemporal aspects of the returning echo and to construct a cohesive and counterfactually rich world model. This complex process involves having predictions about regularities tracking multiple timescales (Kiebel *et al.* 2008), and the differences between the corollary discharge and the input are used to infer properties such as the size, speed and location of the object reflecting the sound. In action sequencing, higher-order corollary discharge is also involved in temporally extended planning strategies—e.g. primates use corollary discharge to keep an internal record of the current saccade to facilitate planning the next saccade (Crapse and Sommer 2008b). Complex reafferent processing is also shown in juvenile songbirds as they imitate the song of tutor (Brainard and Doupe 2000; Margoliash 2002). This requires refining ongoing action plans via continuous updating of an internal record of current state, allowing for flexible contextual interpretation of sensory input towards the realization of temporally deep goals (Crapse and Sommer 2008a).

These complex and context-sensitive sensory filtration mechanisms may be the most promising hallmarks of consciousness in non-human animals. Peter Godrey-Smith—in considering the evolution of subjectivity—reaches a similar conclusion: ‘once animals start to accommodate and utilize reafference, the character of sensing changes. The animal is now not only open to the world, but open to the world as the world, as distinct from self.’ (Godfrey-Smith 2019, 13; Jékely *et al.* 2021). The current account may also provide a fuller picture of why ‘unlimited associative learning’—due to facilitating the complex reafferent processes associated with a deep self-model—may be a marker of the evolutionary transition to minimal consciousness (Bronfman *et al.* 2016; Ginsburg and Jablonka 2019; Birch *et al.* 2020).

## The selflessness challenge

Understanding consciousness in terms of self-consciousness and self-modelling aligns the current account with many other approaches across psychology, neuroscience and philosophy that cast self-consciousness as necessary or constitutive of consciousness itself (Damasio 1999; Metzinger 2003; Gallagher 2010, 2013; Zahavi 2014; Lou *et al.* 2017; Millière 2017). For instance, variations on this claim made in the phenomenological tradition date back at least to Husserl, and more recently Zahavi (2014) says that ‘[S]elf-consciousness is an integral and constitutive feature of phenomenal consciousness […]’ (p. 62). Damasio (1999) argues ‘If “self-consciousness” is taken to mean “consciousness with a sense of self,” then all human consciousness is necessarily covered by the term—there is just no other kind of consciousness as far as I can see’ (p. 19). All these ‘subjectivity theories’ take on the idea that consciousness is phenomenologically centred on the self as the experiencing subject, where consciousness entails a kind of self-consciousness.

Milliere and Metzinger (2020) argues that subjectivity theories are committed to the ‘necessity claim’—the claim self-consciousness is necessary for consciousness in general, in contrast to the ‘typicality claim’—that self-consciousness is merely present in ordinary experience. Millière distinguishes six different notions of self-consciousness that are commonly discussed in the literature, arguing that there is empirical evidence that there are states of consciousness where these states fail to be instantiated.

The necessity claim appears to be embedded into the active inference framework. Friston (2018) states ‘Is self-consciousness necessary for consciousness? The answer is yes. So, there you have it—the answer is yes.’ (p. 1). On one hand, the capacity to formalize the deep links between consciousness and self-consciousness is an explanatory advantage of the active inference framework. It provides a unifying theoretical framework for understanding a host of closely related phenomena, including selfhood, emotion, attention and the sense of agency.

On the other hand, commitment to the stronger claim may present a problem for active inference in delivering a theory of consciousness. Milliere and Metzinger (2020) highlight the fact that this view of self-consciousness as embedded into the very structure of experience may be what Dennett calls Philosopher’s Syndrome: ‘mistaking a failure of imagination for an insight into necessity’ (Dennett 1993, 401). Several recent papers have argued that experiences of altered selfhood present a problem for theories of consciousness that claim self-consciousness is necessary for consciousness (Billon and Kriegel 2016; Letheby 2020; Millière 2020). For instance, Billon and Kriegel (2016) take the cases of ‘inserted thoughts’ in schizophrenia, and the disowned mental states of patients with depersonalization disorder, as problematic cases for proponents of the claim that self-consciousness is necessary for consciousness—as apparent cases where self-consciousness appears to be missing from consciousness.

The subjectivity theorist, then, seems to have two options. The first is to deny that these cases truly present a challenge to subjectivity theories, in virtue of being only ‘partially selfless’. Millière notes that none of the partially selfless states of consciousness would be sufficient to rule out a disjunctive version of the necessity claim—where any form of self-consciousness would be sufficient but not necessary for consciousness. The second option is to deny that there really are states of consciousness lacking in self-consciousness. This line of argument appears more difficult in the face of evidence for states of consciousness that appear to be ‘totally selfless’—lacking in all the ways one could be self-conscious. Both Millière (2020) and Letheby (2020) take the ‘totally selfless’ states of psychedelic-induced ego-dissolution to be evidence against the claim that self-consciousness is necessary for consciousness.

Serotonergic psychedelics such as lysergic acid diethylamide, psilocybin and *N,N*-dimethyltryptamine (DMT; found in ayahuasca) are known to produce profound alterations in phenomenology (Preller and Vollenweider 2016). Most notably for present purposes, psychedelic experiences, especially at high doses, are characterized by profound alterations in self-consciousness (Huxley 1952; Leary *et al.* 1964; Lebedev *et al.* 2015). Both Millière (2020) and Letheby (2020) argue that the ‘total’ ego-dissolution induced by the serotonergic psychedelic 5-methoxy-DMT (5-MeO-DMT) is the strongest evidence against the view that self-consciousness is necessary or constitutive of consciousness. While there is empirical evidence that some advanced forms of meditation practice can also occasion ‘totally’ selfless states (Millière *et al.* 2018; Winter *et al.* 2020; Laukkonen and Slagter 2021), here I will focus on psychedelics as the most robust catalysts of selfless states.

Consider these phenomenological reports of the 5-MeO-DMT experience retrieved from the database of drug experiences erowid.org, cited in Millière (2020) as evidence of ‘totally selfless’ states:

*I was completely disassociated from the ‘* *real world’* *and [from] any sense of self. It was the most jarring feeling. (#107905)*

*It is a complete annihilation of self […]. I was absolutely nothing but a sensory perceiver, stuck within the split seconds that were eternity. (#18198)*

*It felt as if all of the atoms of the molecules that typically form my physical self simply dispersed, and even my sense of self, or ego, vanished […]. (#56384)*

*I wasn’t me any longer. There was no me. There was no ego. (#27601)*

These experiences present considerable counterevidence to the necessity claim, owing to being both vividly phenomenally conscious while totally lacking in any kind of ordinary self-consciousness. Do these experiences provide genuine evidence against the claim that self-consciousness is necessary for consciousness? More specifically, are they problematic for the active inference account of consciousness in terms of self-consciousness? To address this question, the next section will provide an account of ego-dissolution in active inference.

## Psychedelics and selflessness in active inference

The REBUS—‘RElaxed Beliefs Under pSychedelics’—model casts the action of psychedelics in the predictive brain in terms of a ‘relaxation’ (lowering) of the precision of high-level priors, thereby liberating bottom-up information flow (Carhart-Harris and Friston 2020). Although a preliminary account, the REBUS model is growing in empirical support (Alamia *et al.* 2020; Dupuis 2020; Girn *et al.* 2020; Herzog *et al.* 2020). The phenomenology of the psychedelic experience is thought to accord with this description of the underlying computational mechanisms. Recall that, in a predictive coding scheme, if prediction error can be explained away at lower levels, high-level representations of the model remain stable, as there is no need to update. Under psychedelics, the relaxation of high-level priors means that prediction errors that would usually be explained at lower levels are driven up the predictive hierarchy, resulting in instability in higher-level representations, whereby high-level priors no longer constrain lower-level predictions. At lower doses, this manifests as the phenomenological effects of psychedelics—e.g. walls may have the appearance of ‘breathing’ (Carhart-Harris and Friston 2019).

This relaxation of high-level priors results in the system adopting a high Bayesian learning rate on sensory evidence (Mathys *et al.* 2014; Hohwy 2017; Deane 2020). A low learning rate means there is a greater influence of higher-level priors in determining the resulting posterior, and a high learning rate means there is a higher precision on sensory evidence and less constraint imposed by higher-level priors. Appropriately setting the Bayesian learning rate—the precision on sensory evidence—is crucial for the system to approximate Bayesian inference over time, as an overreliance on prior expectations leads to a failure to learn from sensory evidence and an overreliance on sensory evidence can lead the system to ‘overfit’—essentially, find patterns in noise. The perceptual effects of psychedelics have been characterized as ‘rampant’ overfitting of sensory evidence (Deane 2020)—where the system cycles through candidate hypotheses to explain the influx of highly precise prediction error ascending the cortical hierarchy.

Another feature of psychedelic phenomenology is that sensory impressions often take on a unique significance often described as highly aesthetic. Consider Aldous Huxley’s descriptions of the mescaline experience in ‘The Doors of Perception’ (1952):

*I looked down by chance, and went on passionately staring by choice, at my own crossed legs. Those folds in the trousers—what a labyrinth of endlessly significant complexity! And the texture of the gray flannel—how rich, how deeply, mysteriously sumptuous!* (p. 39)

*The books, for example, with which my study walls were lined. Like the flowers, they glowed, when I looked at them, with brighter colors, a profounder significance.* (p. 24)

On the current account, this quality of significance accompanying the perceptual effects of psychedelics can be understood as the system inferring high epistemic value due to the high precision on sensory evidence. This is because ‘the better the precision on the prediction error, the higher the learning rate; that is, the more we trust the quality of the evidence the more we should learn from it’ (Hohwy 2017, 76). Under the view of affective experiences broadly described earlier as inference about ‘how well am I self-evidencing?’—the positive emotions in psychedelic experiences: ‘exhilarated elation with unmotivated laughter, deep feelings of peace, exuberant joy, and hedonistic pleasure’ (Preller and Vollenweider 2016, 236)—could put down to the greater than expected epistemic value associated with the current policy. This point is relevant for the affective characterization of ego-dissolution to come.

### Psychedelic-induced ego-dissolution

Deane (2020) characterizes psychedelic-induced ego-dissolution as resulting from a failure of sensory attenuation (see Girn *et al.* 2020; for recent empirical support for this hypothesis). Recall that predictions of the sensory consequences of actions (‘corollary discharges’) allow the system to differentiate between endogenous (self) and exogenous (other) causes of sensation, such that unexpected sensation is attributed to external causes. Under a view of phenomenal selfhood as an allostatic control model, the sense of being an agent arises from inferring oneself to be an endogenous cause of sensation; i.e. determined by the predictability of action-outcome contingencies. A number of disruptions in self-experience have been accounted for in these terms. For instance, the symptomatology of schizophrenia—such as thought insertion, where patients report feeling that their thoughts are not their own—have been understood in terms of a failure of these sensory attenuation mechanisms (Ford and Mathalon 2005; Frith 2005; Rösler *et al.* 2015; Thakkar *et al.* 2021). Here, the system fails to attribute self-generated outcomes to endogenous rather than exogenous causes, with the phenomenological manifestation to the agent being a perceived loss of agency over their thoughts (Stephens and Graham 1994; O’Brien and Opie 2003; Gallagher 2004). In the case of voice hearing this can result in attribution of inner speech to an external source such as another agent (Ford and Mathalon 2005; Ford *et al.* 2007).

Corollary discharges—as predictions of the sensory consequences of actions—act to cancel out self-generated sensory outcomes via sensory attenuation. Unexpected consequences are then attributed to exogenous rather than endogenous causes. This means that the more sensory prediction error is generated, the more likely it is that an action or thought has external as opposed internal or endogenous causes (Frith 2005; Corlett *et al.* 2019). Deane (2020) notes that under the REBUS model of the action of psychedelics, the influx of both exteroceptive and interoceptive prediction error means that the outcomes of actions (and mental actions) become radically unpredictable. As a result, the system ceases to posit itself as an endogenous controller of sensation (and as a causally efficacious agent)—manifesting phenomenologically as ego-dissolution. In the account of thought insertion above, the thought was attributed to ‘other’ rather than self, due to not being inferred to be self-generated, based on a failure of these mechanisms. Ego-dissolution here is being understood in terms of similar mechanisms to the example of thought insertion described above but is experienced as a more global dissolution of selfhood due to the influx of unpredictable inputs from across the cortex, as opposed to being isolated to certain activity.

### Affective tone

While ego-dissolution is described as being devoid of self-consciousness, it is nonetheless described as a highly conscious state, characterized by affective extremes. Carhart-Harris and Friston (2020) distinguish between ‘complete ego-dissolution’—a state of ‘complete surrender, associated bliss, and union with all things’ (Carhart-Harris and Friston 2019, 321); and ‘incomplete’ ego-dissolution—a state characterized by intense fear, anxiety and distress.

‘Complete’ ego-dissolution can be understood on the current account to be underpinned by two closely related computational mechanisms. The first relates to pragmatic value and prior preferences. Recall that, on the account of self-modelling proposed here, the inference on allostatic control tunes precision on expected free energy. For instance, this could be a higher precision on a particular prior preference (consider the example of pain perception given earlier). Precision on unfulfilled prior preferences can be a persistent source of suffering to the system—one such example being chronic pain, where chronic pain is underpinned by high precision on a prior preference that is unable to be fulfilled through action (Hechler *et al.* 2016). On the view that action arises from minimizing the discrepancy between the actual (inferred) current state and the desired state, relaxation of the constraining influence of high-level priors means they cease to structure consciousness to engage the organism in their fulfilment, and as such, end their associated suffering. High-level priors constraining more domain-general affective states such as mood (Clark *et al.* 2018) would also be relaxed under the REBUS model. This connects closely to the therapeutic potential of the experience: ‘psychedelics work to relax the precision weighting of pathologically overweighted priors underpinning various expressions of mental illness’ (Carhart-Harris and Friston 2020, 1). Deane (2020) highlights that the lessened influence of prior preferences accords with descriptions of the phenomenology of ego-dissolution, for instance: ‘It felt as if “I” did no longer exist. There was purely my sensory perception of my environment, but sensory input was not translated into needs, feelings, or acting by “me”’ (unpublished online survey data quoted in Millière *et al.* 2018, 7).

There is another reason ‘complete’ ego-dissolution may be characteristically ecstatic. Inference about allostatic control is not just about realizing the pragmatic affordances of action, but also in maximizing the epistemic value associated with a given policy. Recall that, in normal functioning, precision on sensory information would track the expected epistemic value of sensory inputs. In the psychedelic state, the relaxation of high-level priors, and corresponding increase in sensory precision, means the system infers that the current state is realizing great epistemic value (See ‘Psychedelics and Insight’ in Carhart-Harris and Friston, 2020). This particular phenomenological quality seems unique to the serotonergic psychedelics—in a direct comparison between psilocybin and dextromethorphan (a non-serotonergic psychedelic) experiences, psilocybin produced ‘greater visual, mystical-type, insightful, and musical experiences’ (Carbonaro *et al.* 2018, 1).

## Responding to the selflessness challenge

We can now return to the question of whether psychedelic-induced ego-dissolution threatens active inference theories of consciousness grounded in self-modelling. To recap the central position of this paper, subjectivity is underpinned by an inference about allostatic control—namely an inference on ‘how well am I self-evidencing’. In other words, subjectivity is underpinned by an inference evaluating about how well the system is bringing about (phenotype-specific) expected outcomes, informing policy selection, and shapes subjectivity determining what is salient to the organism. This hierarchically deep inference about precision on the action model is understood to be the computational underpinnings of phenomenal selfhood—the sense of being an agent. Phenomenal selfhood on this view is deep in that it relates to an inference about the fit between actual and expected outcomes on multiple hierarchical levels, tracking expected outcomes on proximal and distal timescales. In other words, this evaluation of the fit between the expectations and outcomes ranges from transient sensorimotor expectations about the how sensation will unfold contingent on motor actions, to the temporally deep (and abstract) expectations of allostatic control across contexts. These ‘deep’ self-models structure subjectivity in permeating perception of the world—the ‘meaning’ of sensation is determined by what it means ‘for me’—i.e. the implications for control of interoceptive states and allostatic control.

As we have seen, at first glance selfless states such as those occasioned by psychedelics threaten the necessity relationship between phenomenal self-modelling and consciousness. However, equipped with the account of the computational mechanisms underpinning psychedelic-induced ego-dissolution of the previous section, the inferential process evaluating the fit between model and world is shown to be intact, as the phenomenology of ego-dissolution (complete or incomplete) on this account is underpinned by a very particular inference about allostatic control that remains present in the psychedelic state.

The present account puts subjective valuation as the most basic constitutive feature of a conscious experience—sensation is always infused with what it ‘means’ for the organism: ‘not everything that happens to us enters our awareness, not by far … but everything that does is not merely informationally registered but also felt’ (Kolodny *et al.* 2021, 2). This fact is brought into sharp relief in the account of psychedelic-induced ego-dissolution, as affective experience remains even when all the other structuring features of experience are extinguished. The upshot of this view is that affective valence can be understood as the most fundamental part of conscious experience (Panksepp 1998, 2005, 2008; Damasio 1999, 2018; Damasio and Carvalho 2013; Man and Damasio 2018). This is a view that dates back at least as far as George John Romanes

‘The raison d’être of Consciousness may have been that of supplying the condition to the feeling of Pleasure and Pain.’ (Romanes 1888, 111)

Grounding consciousness in basic affectivity also has a precedent in the literature on the free energy principle and consciousness, which is particularly consonant with the current picture. Mark Solms has argued, partially based on evidence of consciousness in decorticated animals and congenitally decorticate (hydranencephalic) humans, that

‘Consciousness itself is affective. Everything else (from motivation and attention, leading to action and perception, and thereby to learning)—all of it—is a functional of affect. Affect obliges the organism to engage with the outside world.’ (Solms 2019, 12)

Moreover, this view seems aligned with a view of ego-dissolution as only partially selfless states and with the view proposed here that higher layers of the phenomenal self-model structure consciousness

‘Affect just is a self-state (and through feeling—i.e., precision optimisation—it necessarily generates consciousness itself), which activates (selects) salient perceptual representations, which eventually include cognitive re-representations of the self.’ (Solms and Friston 2018, 17)

On this view, feelings in the form of subjectively felt valence are the most basic constitutive phenomenal states, they pervade all of experience, guiding the organism to fitness-promoting states (Inzlicht *et al.* 2015; Kolodny *et al.* 2021). While in typical experience, subjective valuation functions to fine-tune learning (Eldar *et al.* 2016) and regulate behaviour, we can see the same mechanisms in place in atypical experience such as the psychedelic state.

It is reasonable to assume that many will not be satisfied with this characterization of self-consciousness. For instance, on the definition of self-consciousness as consciousness of oneself ‘as oneself’ (Smith 2017; Millière 2020), it seems reasonable to conclude that psychedelic-induced ego-dissolution is best understood as being totally selfless, as argued by Millière (2020) and Letheby (2020). It may be the case that whether ego-dissolution is understood as a state totally devoid of self-consciousness boils down to how self-consciousness is defined. Millière (2017, 2020) notes that the disagreements about the necessity claim and the typicality claim may hinge on terminological variation (Guillot 2017), due to the polysemy of ‘self-consciousness’ and ‘sense of self’. For present purposes, this is inconsequential. On its more stringent definition, it may be argued that self-consciousness is truly absent in states of drug-induced ego-dissolution—i.e. if the affective inference present in these states is deemed not to qualify as self-consciousness due to not being understood as a representation of oneself ‘as oneself’. On this definition, the active inference approach to consciousness put forward in this paper simply does not qualify as a subjectivity theory and so is not vulnerable to the selflessness challenge.

It is worth acknowledging there that while psychedelic states may instantiate affective consciousness in the absence of sensorimotor consciousness, the current account does not rule out that there can be states of consciousness that vary on these dimensions. The possibility of states of consciousness, which in contrast to the psychedelic state, score low on domain-general affectivity and high on sensorimotor consciousness is consistent with the current account. Godfrey-Smith (2019) argues that sensorimotor consciousness and evaluative consciousness may be separable dimensions of consciousness, raising the possibility of two types of phenomena that are grouped under ‘subjective experience’:

‘If we ask, introspectively, about conspicuous features of human experience that may have early forms, it might be intuitive that one side of the phenomenon involves tracking external objects and events as external – achieving a point of view on things – while another involves distinctions between good and bad, a distinction that might be present in phenomenal washes that have no definite referral to organism or to environment.’ (p. 14)

Godfrey-Smith goes on to note that some spiders demonstrate complex perceptual capacities, but score low in respect to evidence for complex or varying motivational states. This would be expected in creatures that, given an evolutionary niche, do not need a more sophisticated ‘domain general controller’ instantiated by affective inference. Other creatures—such as certain gastropods—may have richer subjective valuation in the absence of more complex sensorimotor capacities. On the current account, however, both sensorimotor and evaluative aspects of consciousness are underpinned by a common evaluative inference about realizing phenotype-congruent outcomes across multiple timescales. In other words, an inference about how well the organism is bringing about expected states from low-level and temporally proximal motor plans to high-level and temporally distal expectations of self-evidencing outcomes. It is therefore consistent with the present account that the sensorimotor and affective aspects of consciousness can vary independently. That said, on the present account, sensorimotor consciousness is at least minimally affective owing to being driven by ‘systematic misrepresentations’ of the system in preferred states of being—it arises through the system tracking the discrepancy between actual and preferred states of being.

The active inference approach to consciousness and subjectivity put forward in this paper casts consciousness and self-consciousness as intimately connected, where phenomenal self-models ‘shape’ subjectivity. However, I have aimed to show that this approach can also accommodate (and illuminate) phenomenological states largely lacking in self-consciousness. These ‘selfless states’—understood as ‘(rare) cases in which normally congruent processes of computational and phenomenal self-modelling diverge’ (Limanowski and Friston 2020, 12)—retain the core aspect of phenomenal self-modelling—subjective evaluation—even in drastically altered states of consciousness like ego-dissolution.

## Conclusion

This paper has argued that phenomenal consciousness is best understood within predictive processing in terms of the deep self-models inherent in the active inference framework. On this account, subjectivity is structured by a ‘deep control model’—a hierarchically deep self-model that is tracking the temporally deep endogenous control of self-evidencing outcomes. Higher levels provide deep contextualization (interoceptive inference) of afferent signals from the body (Miller and Clark 2017), tuning the organism to adaptive opportunities for action. Two objections to this view have been considered: (i) that the core characteristics of consciousness in predictive processing is underspecified and as such cannot inform which systems are conscious, and (ii) the challenge of psychedelic-induced ego-dissolution. I have argued that neither of these objections is troubling for an active inference theory of consciousness and as such active inference is a very promising framework for consciousness science.

## Data Availability

None declared.
